# Influence of Dehydration Temperature on Obtaining Chia and Okra Powder Mucilage

**DOI:** 10.3390/foods12030569

**Published:** 2023-01-28

**Authors:** Francislaine Suelia dos Santos, Rossana Maria Feitosa de Figueirêdo, Alexandre José de Melo Queiroz, Yaroslávia Ferreira Paiva, Henrique Valentim Moura, Eugênia Telis de Vilela Silva, João Paulo de Lima Ferreira, Bruno Adelino de Melo, Ana Júlia de Brito Araújo Carvalho, Marcos dos Santos Lima, Caciana Cavalcanti Costa, Wilton Pereira da Silva, Josivanda Palmeira Gomes

**Affiliations:** 1 Department of Agricultural Engineering, Federal University of Campina Grande, Campina Grande 58429-900, Brazil; 2 Department of Process Engineering, Federal University of Campina Grande, Campina Grande 58429-900, Brazil; 3 Federal Institute of Sertão Pernambucano, Department of Food Technology, Petrolina 56314-522, Brazil

**Keywords:** food drying, bioactive compounds, antioxidant activity

## Abstract

Gum and mucilage from seeds and fruits are objects of study because they have characteristics of high viscosity at low concentrations and gelling properties, which are useful characteristics for modifying the texture and stabilizing products in the food industry. Chia and okra have high concentrations of polysaccharide gums in their composition, which makes them an interesting target for use in the composition of foods that require the use of texture enhancers and stabilizers. The present study investigated the influence of dehydration temperature on the characteristics of chia and okra powder mucilage obtained at different temperatures. The mucilages were extracted using an aqueous process and dehydrated in an air circulation oven at 50, 60, and 70 °C until hydroscopic equilibrium. Then, the powdered chia mucilage (CM) and okra mucilage (OM) were analyzed for chemical and physicochemical characteristics, bioactive compounds, antioxidant activity, and physical properties. It was found that powdered mucilage had low water content and water activity, with CM standing out in terms of ash, pectin, and starch content and OM, along with higher averages of proteins, sugars, total phenolic compounds, anthocyanins, flavonoids, and antioxidant activity. As for the physical parameters, CM stood out in relation to greater solubility and lower hygroscopicity, whereas OM presented higher wettability rates. Both powdered mucilages were classified as having good fluidity and cohesiveness from low to intermediate. In relation to the dehydration temperature, the best mucilage properties were verified at 70 °C. The study revealed that mucilages have good functional properties offering great potential as raw material for industry.

## 1. Introduction

Mucilage is a hydrocolloid composed of long-chain polymers with high molecular weight, polysaccharides, and proteins, which have a great affinity with water, being partially or totally soluble, where they disperse forming viscous solutions. They are extracted from grains or seeds (chia and linseed), from plants and pods (okra), from algae (agar, alginate and carrageenan), and from animals (gelatin and chitin) [1,2].

Chia seed (*Salvia hispanica*), belonging to the genus Salvia, is known for its high concentration of alpha linolenic acid and omega 3; it also has dietary fiber, proteins, lipids, carbohydrates, vitamins, minerals, and phenolic compounds in its composition [3,4]. When in contact with water, it releases a mucilaginous gel of high solubility and with thickening properties, rich in antioxidants, proteins, soluble fiber, and polyunsaturated fatty acids; its gum is isolated from the seed coat, depending on the varieties and growing conditions, and it constitutes about 4–6% of the seed dry mass [1,5]. Chia proteins favor its emulsifying properties, while its high carbohydrate and fiber content favors gel formation [6]. Chia has numerous benefits for industries and consumers, being beneficial to health, and it can be used in numerous applications in the food and pharmaceutical areas. Among the possible applications, the use as a wall material in spray-drying and microencapsulation [5], formulation of edible films [7], stabilization of emulsions [8], and nanoencapsulation by lyophilization [6] stand out, among others. Okra (*Abelmoschus esculentus* (L.) Moench) is a vegetable that represents an important source of antioxidants and nutrients, such as polyphenols, minerals, vitamin C, and polysaccharides such as pectin and lignin [9]. Its mucilage consists mainly of pectin, which is a polysaccharide formed by galactose, rhamnose, and galacturonic acid units [10]. The use of okra mucilage as an emulsifying agent in acid environments has shown good results, including with regard to stability. Therefore, its use has a high potential in fruit and dairy drinks, which generally have an acid nature and can be applied in the form of carriers, emulsifiers, stabilizers, and encapsulating agents [9,11,12] in the manufacture of films, among other uses.

The use of these mucilages has advantages over synthetic polymers due to the lower acquisition cost and their biodegradability, and they can be applied as a thickener, stabilizing emulsion, suspending agent, and binder in the food and pharmaceutical industries. Mucilage dehydration processing has the potential to make them available for a prolonged period, presenting itself as an alternative to increase the product useful life, to concentrate the nutrients, and to facilitate the industrial application, transport, storage, and commercialization [13].

The chemical composition of hydrocolloid varies greatly depending on the type of material extraction, processing method, and temperature, as well as according to its origin. Although some studies have been carried out to evaluate and characterize chia and okra mucilage [1,14,15,16,17]. There is great variability between the isolation methods and the yields achieved, implying discrepancies in the chemical constitution and functional attributes of the resulting polysaccharide. In this sense, the present work was carried out with the objective of extracting the mucilage of chia and okra, obtaining the mucilages in powder from the convective drying at different temperatures (50, 60, and 70 °C), and evaluating the influence of the dehydration temperature on the chemical and physicochemical characteristics, bioactive compounds, antioxidant activity, and physical properties of the powders.

## 2. Materials and Methods

### 2.1. Raw Materials and Processing

The raw materials used were chia seeds (*Salvia hispanica*) and the Santa Cruz variety of okra (*Abelmoschus esculentus* (L.) Moench) in the green maturation stage, obtained from local businesses in the city of Campina Grande, PB, in the summer season.

In the laboratory, the okras were selected manually, in order to eliminate those with physical damage or at another stage of maturation; then, they were washed in running water, immersed in containers containing sodium hypochlorite solution at a concentration of 50 ppm, for 15 min, and finally rinsed in running water.

### 2.2. Extraction of Mucilage and Obtaining the Powders

#### 2.2.1. Chia Mucilage

Chia mucilage was extracted following the methodology of Muñoz et al. [18] including adaptations according to the patent report BR 10 2020 007595 0. The seeds were immersed in distilled water at a ratio of 1:15 for 30 min, followed by crushing with a domestic blender (Arno, model Power max 1000 W, Itapevi, São Paulo, Brazil) at maximum speed for 1 min, before being placed in a water bath at 80 °C, stirring for 2 h. The mixture was filtered through organza (woven polyester fabric consisting of twisted fibers) and transferred to a glass container. Hydrated ethanol (92° INPM) was added in the proportion 1:2 according to the tests carried out, until the precipitation of solids. Then, the mixture was subjected to agitation, left to rest at 4 °C for 24 h, and, after separating (decanting) the phases, the solvent (ethanol) was drained off through organza filtration, obtaining chia mucilage (solute).

#### 2.2.2. Okra Mucilage

The steps of the okra mucilage extraction process were carried out according to the methodology of Muñoz et al. [18] with adaptations. Initially, the okras were cut into slices; distilled water was added in a ratio of 1:5, before crushing in a blender (Arno, model Power max 1000 W, Itapevi, São Paulo, Brazil) at maximum speed for 1 min. The sample was placed in a water bath at 80 °C, stirring for 2 h; then, the mixture was filtered into organza and transferred to a glass container. Hydrated ethanol (92° INPM) was added in the proportion 1:1, according to the tests carried out, until the precipitation of solids. The mixture was subjected to agitation and allowed to rest at 4 °C/24 h. After the separation (decantation) of the phases, the solvent (ethanol) was drained through organza filtration, obtaining okra mucilage (solute). The adaptations made in the extraction process of okra mucilage were deposited in patent report BR 10 2020 00844 4.

#### 2.2.3. Obtaining Powdered Mucilages

After the extractions, the chia and okra mucilages were homogeneously placed in stainless-steel trays and subjected to convective drying in an oven with forced air circulation (Fanem, model 320, Guarulhos, São Paulo, Brazil), at temperatures of 50, 60, and 70 °C and an air velocity of 1.0 m s^−1^, performing regular weight checks using a semi-analytical balance (Marte, model AS5500C, Santa Rita do Sapucaí, Minas Gerais, Brazil), until reaching the hygroscopic equilibrium. Then, the dehydrated mucilage was crushed in a knife mill (Marconi, model TE 340, Piracicaba, São Paulo, Brazil) to obtain the powders.

### 2.3. Chemical and Physicochemical Characterization

The powdered mucilages obtained were characterized, in quadruplicate, according to the parameters described below.

#### 2.3.1. Yield

The mucilage powder yield (R%) was determined by the ratio between the initial (before drying) mucilage mass (M_i_) and the final powder mucilage mass (M_f_) (Equation (1)) [19].

(1) $$R\;(\%)=\frac{M_{f}\;\text{×}\;100}{M_{i}}$$

#### 2.3.2. Water Content, Total Titratable Acidity, Total Soluble Solids, Ash, and Total Proteins

The water content was determined using the standard greenhouse method (New Lab, model NL 82-27, Piracicaba, São Paulo, Brazil) at 105 ± 3 °C, until reaching a constant mass. The total titratable acidity was determined by the acidimetric method, using 0.1 M sodium hydroxide solution, with the results expressed as a percentage of citric acid. The total soluble solids (°Brix) were determined by the refractometric method, in an Abbé type refractometer (Instrutherm, modelo RT–30 ATC, São Paulo, SP, Brazil), with the results expressed in °Brix. The ash content was determined by calcination of the sample in a muffle (Forlabo, modelo BioFM 6.7 L, Diadema, São Paulo, Brazil) at 550 ± 5 °C. The total proteins were quantified by the determination of nitrogen performed through the micro-*Kjeldahl* method, comprising three steps (acid digestion, distillation, and titration). All of the aforementioned determinations were carried out in accordance with the methodologies recommended by the AOAC [20].

#### 2.3.3. Water Activity

The water activity was determined by direct reading of the sample in a dew point hygrometer (Aqualab, model 3TE, Decagon, Pullman, WA, USA) at the temperature of 25 °C.

#### 2.3.4. pH

The pH was determined by the potentiometric method, with a pH meter (Tecnal, model TEC-2, Piracicaba, São Paulo, Brazil), previously calibrated with pH 4.0 and 7.0 buffer solutions.

#### 2.3.5. Sugars

The total sugars were determined using the methodology described by Yemm and Willis [21], in which the samples were submitted to analysis in a spectrophotometer (Coleman, model 35-D, Santo André, São Paulo, Brazil) at 620 nm, and the quantification of sugars was based on a glucose standard curve.

Reducing sugars were determined by Miller’s methodology [22], using 3,5-dinitrosalicylic acid (DNS) as the oxidizing agent, with spectrophotometer (Coleman, model 35-D, Santo André, São Paulo, Brazil) readings at 540 nm.

#### 2.3.6. Starch

The determination of total starch was performed by spectrophotometry (Coleman, model 35-D, Santo André, São Paulo, Brazil) at 620 nm, monitoring the colored compound formed by the reaction between anthrone and glucose from starch hydrolysis [23].

#### 2.3.7. Pectin

The pectin content was determined using Pearson’s methodology [24], by neutralizing the charges of free uronic residues by calcium ions, causing pectin to gel and precipitate.

### 2.4. Bioactive Compounds

#### 2.4.1. Total Phenolic Compounds

Total phenolic compounds were quantified using the Folin–Ciocâlteau method described by Waterhouse [25]. The extracts were obtained with 1.0 g of the sample macerated with 50 mL of distilled water, followed by resting for 30 s, and then filtered through filter paper. Then, an aliquot of the extract, distilled water, and Folin–Ciocâlteu reagent were homogenized in a vortex (E-Labcommerce, Model XH-CU, Monte Alto, São Paulo, Brazil), and left for 5 min. To this mixture, 250 μL of sodium carbonate solution (20%) as added, followed by vortexing and heating in a water bath at 40 °C for 30 min in the absence of light during the test. After reading on a spectrophotometer at 765 nm (Coleman, model 35-D, Santo André, São Paulo, Brazil), the results were expressed as mg EAG (gallic acid equivalent) per 100 g db.

#### 2.4.2. Tannins

Tannins were determined according to the methodology described by Goldstein and Swain [26] using a tannic acid curve as a standard. The extracts were obtained with 0.5 g of the sample macerated with 25 mL of distilled water, followed by resting for 30 s, and then filtered through filter paper. Then, an aliquot of the extract, distilled water, and Folin–Ciocâlteu were homogenized in a vortex (E-Labcommerce, Model XH-CU, Monte Alto, São Paulo, Brazil) and rested for 5 min; to this mixture, 250 μL of sodium carbonate solution (20%) was added, followed by vortexing and heating in a water bath at 40 °C for 30 min in the absence of light during the test. Next, readings were performed in a spectrophotometer at 765 nm (Coleman, model 35-D, Santo André, São Paulo, Brazil), and the results were expressed as mg EAT (tannic acid equivalent) per 100 g db.

#### 2.4.3. Total Anthocyanins and Flavonoids

Total anthocyanins and flavonoids were determined by spectrophotometry (Coleman, model 35-D, Santo André, São Paulo, Brazil) according to the methodology described by Francis [27]. The extract was prepared with 0.5 g of the sample and 25 mL of ethanol–HCL solution (1.5 N) in the proportion of 85:15, homogenized in a magnetic stirrer (FANEM, model 257, Guarulhos, São Paulo, Brazil), placed in a refrigerator (8 ± 1 °C) for 24 h, and then subjected to filtration on filter paper (80 g/m^2^). Absorbance readings were performed at 535 nm for anthocyanins and 374 nm for flavonoids; the results were expressed as mg/100 g db.

#### 2.4.4. Antioxidant Activity

Antioxidant activity was evaluated using three methods: the iron reduction method (FRAP—ferric reducing antioxidant power) and ABTS (2,2-azinobis 3-ethylbenzthiazoline-6-sulfonic acid), both described by Rufino et al. [28], and the elimination of free radicals by DPPH (2,2-diphenyl-1-picrylhydrazyl) according to the methodology described by Morales and Jiménez-Pérez et al. [29], in which the transfer of electrons and hydrogen atoms was detected. The Trolox analytical standard (6-hydroxy-2,5,7,8-tetramethylchroman-2-carboxylic acid) was used to construct the calibration curves, except for the FRAP method, in which ferrous sulfate was used as the standard. Results were expressed as Trolox equivalent per kg of mucilage powder (mmol ET/kg db) and mmol Fe^2+^ per kg of mucilage powder (mmol Fe^2+^/kg db).

To determine the antioxidant activity, the samples were diluted in deionized water at a ratio of 1:10. Absorbance readings were performed in a UV/visible spectrophotometer (model UV 2000a, Instrutherm, Brazil).

In the DPPH assay, the extract of each mucilage (100 μL) was mixed with a solution of 2.9 mL of DPPH (1 mM/L), prepared in absolute ethanol, and diluted to 0.900 ± 0.080 (100 μmol/L) of absorbance. The antioxidant activity of the samples was determined by reading the decay rate at 517 nm, performed in a spectrophotometer, determined 30 min after adding the sample.

For the ABTS method, the antioxidant activity of each mucilage was determined by the formation of the ABTS radical, formed by the reaction of 5 mL of ABTS (7 mM) with 5 mL of potassium persulfate (2.45 mM) in a dark environment, for 16 h at 25 °C. The ABTS solution was diluted with 80% ethanol to adjust the absorbance between 0.700 ± 0.050, with the decay rate reading at 734 nm. Then, 0.5 mL of the sample extract was added to 3.5 mL of the ABTS radical solution and read in a dark environment at time 0 and after 6 min of reaction.

In the FRAP method, to prepare the FRAP reagent, 25 mL of acetate buffer solution (300 mM/L; pH 3.6) was combined with 2.5 mL of FeCl_3_ (20 mM) and 2.5 mL of TPTZ solution (10 mM/L 2,4,6tris(2-pyridyl) triazine in 40 mM HCl). A 90 μL aliquot of the powdered mucilage extract was diluted in 2.7 mL of FRAP reagent and placed in a thermostatic bath (Bio plus, model IT2002, Barueri, Brazil) at 37 °C for an incubation period of 30 min. Absorbance was determined in a spectrophotometer at 595 nm, using the FRAP solution as a control.

### 2.5. Physical Characterization

#### 2.5.1. Hygroscopicity

Hygroscopicity was determined according to the methodology proposed by Cai and Corke [30]; the results were expressed as g of adsorbed water per 100 g of sample mass.

#### 2.5.2. Solubility

Solubility was determined according to the method described by Cano-Chauca et al. [31]. Briefly, the sample was centrifuged in a centrifuge (QUIMIS, model Q222TM2, Diadema, Brazil) at 3200 rpm for 15 min; then, the supernatant was removed and placed in a previously tared Becker, in an oven (New Lab, model NL 82-27, Piracicaba, São Paulo, Brazil) at 105 °C/24 h, for the determination of the soluble mass.

#### 2.5.3. Wettability

The wettability was determined using the Schubert method [32], expressed as the ratio between the mass (g) and the time required for the sample to disappear from the surface (min).

#### 2.5.4. Apparent and Compacted Density

The apparent density (ρ_ap_) was determined with the aid of a 10 mL test tube previously weighed and later filled with the powder, according to the mass/volume ratio. The compacted density was determined from the assembly used in the apparent density; the cylinder filled with the sample was subjected to 50 beatings on the bench, from a pre-established height of 2.5 cm, calculating the relationship between compacted mass and volume [33].

#### 2.5.5. Carr Index and Hausner Factor

The Carr index (CI) and the Hausner factor (FH) were determined using the methodology of Santhalakshmy et al. [34], in which these values were calculated from the apparent density (ρ_ap_) and compacted density (ρ_c_) data, according to Equations (2) and (3).

(2) $$\mathrm{IC}=\frac{\;\rho_{c}\;–\;\rho_{\mathrm{ap}}}{\rho_{\mathrm{ap}}}100$$

(3) $$\mathrm{FH}=\frac{\rho_{c}}{\rho_{\mathrm{ap}}}$$

### 2.6. Statistical Data Analysis

Data were analyzed using variance (ANOVA) in a 2 × 3 factorial scheme, with two powdered mucilages (chia and okra) and three drying temperatures (50, 60, and 70 °C). The comparison between means was performed by applying Tukey’s test at 5% probability, using the Assistat version 7.7 beta computer program [35].

## 3. Results and Discussion

### 3.1. Chemical and Physicochemical Characterization of Powdered Mucilages

Table 1 shows the chemical and physicochemical parameters of powdered chia (CM) and okra (OM) mucilages obtained at temperatures of 50, 60, and 70 °C.

After drying, CM showed a statistically higher yield (*p* < 0.05) than OM at all analyzed temperatures. Considering the OM, there was a statistical difference between the dehydration temperatures, with 50 °C exhibiting the highest value followed by 60 and 70 °C, showing a reduction of 27.39% from the highest to the lowest temperature. As for CM, there was a similar behavior, but there was no statistical difference between the temperatures of 50 and 60 °C and an average reduction of 18.92% at the temperature of 70 °C.

Lower results were reported by Felisberto et al. [14], who obtained a drying yield of 7.86% for lyophilized chia mucilage; Capitani et al. [8] verified a yield of 3.8% for chia mucilage obtained by the lyophilizing method. Antigo et al. [15], studying the influence of drying methods (drying in an oven at 50 °C and lyophilizing) on the centesimal composition of the chia seed and psyllium husk mucilages, obtained a drying yield of 4.38% and 5.86% for chia seeds and 52.11% and 55.45% for psyllium husk in the respective dehydration methods. Gemede et al. [16], when analyzing okra powder mucilages (dehydrated at 45 °C in a circulation oven), verified a yield variation from 1.25 to 3.45 g/100 g. Lastly, Araújo et al. [17] obtained a yield of 1.67% for the mucilage of lyophilized and without seeds okra.

The water content of CM was lower than that of OM at all temperatures studied, with a statistical reduction (*p* < 0.05) in the parameter being verified with a temperature increase in both materials. CM showed a reduction in water content of 57.85% from the lowest to the highest temperature. On the other hand, OM obtained a smaller reduction with the temperature increase, being 24.94% from 50 to 70 °C. Timilsena et al. [36], when lyophilizing chia mucilage extracted in a ratio of 1:20, reported a water content of 4.05% db. Araújo et al. [17] found for powdered okra mucilage, obtained from lyophilizing and from seedless extraction in the ratio of 1:2.5 (okra to water), a water content of 0.452% db. Pontes et al. [37], extracting mucilage from chia (*Salvia hispanica* L.) and from brown linseed (*Linum usitatissimum* L.) in a 1:25 ratio, followed by drying in an oven with air circulation at 45 °C, obtained water contents of 4.96% and 7.79% db, respectively.

As with the water content, CM showed a lower water activity than OM at all temperatures under study. There were statistically significant reductions (*p* < 0.05) in both materials with increases in drying temperature, probably due to the lower water content of samples at high temperatures. The reductions in water activity of powdered chia and okra mucilage from 50 to 70 °C drying temperature were on the order of 50.00% and 41.45%, respectively. According to Resende et al. [38], low water activities (aw < 0.600) confer stability during storage, as they are considered microbiologically stable; this reasoning can be applied to the powdered mucilages obtained in this study.

For the ash content, there was a statistical difference (*p* < 0.05) between the materials, highlighting CM with the highest averages in all drying temperatures. Among the powdered mucilages, there was no statistical difference (*p* < 0.05) with the dehydration temperature increasing. Both mucilages had lower ash levels than commercial mucilages, such as gum Arabic (20.26% db), carrageenan gum (25.45% db), and xanthan gum (12.00% db) [39]. Pontes et al. [37] verified higher ash contents of 7.90% and 4.76% db, respectively, for the mucilage of chia (*Salvia hispanica* L.) and brown flaxseed (*Linum usitatissimum* L.) powder obtained in an oven with air circulation at 45 °C.

Powdered mucilages showed low acidity, with OM standing out with lower values. It was verified that, in CM, there was no statistical difference (*p* < 0.05) with the increase in drying temperature, while, in OM, there was a reduction of 5.62% from the lowest to the highest dehydration temperature, with no statistical difference between the temperatures of 60 and 70 °C. According to Melo et al. [40], this reduction is probably due to the thermal degradation of the organic acids as a result of the total titratable acidity, encompassing all the acids present in the product, assuming that any loss verified in any of the constituent acids would interfere with these results. Carmona, Robert, and Sáenz [41] also verified low acidity when studying the effect of the spray drying process on the properties of the mucilage extracted from *Opuntia ficus-indica* Cladodes, obtaining values of 0.003% citric acid before drying and 0.002% citric acid after reconstitution.

The pH of powdered mucilages was close to neutrality, with values greater than 6.0, highlighting CM with higher values (*p* < 0.05). It was found that, with the increase in drying temperature, there was a tendency to increase pH values in both materials. The inverse correlation between pH and total titratable acidity was verified in all samples, corroborating the heating effect on both. A similar pH value in dehydrated mucilage was reported by Pereira et al. [42], who, when studying the extraction of mucilage from mutamba fruits (*Guazuma ulmifolia* Lam.), verified a pH of 6.6 for the mucilage dehydrated in an oven with air circulation at a temperature of 40 °C.

Protein contents were statistically higher (*p* < 0.05) in OM at temperatures of 50 and 60 °C and statistically lower at 70 °C in relation to CM. The OM presented a reduction of about 25.88% with the increase in the drying temperature, from the lowest to the highest temperature, while the CM remained statistically stable. Lower values were reported by Felisberto et al. [14] for powdered chia mucilage, extracted at a ratio of 1:40 followed by lyophilizing at 12.33% db. Zim et al. [43], when studying okra mucilage dehydrated in a circulation oven at 45 °C, found a content of 8.54 g/100 g db.

CM showed higher mean pectin contents compared to OM at all temperatures under study, with statistical differences (*p* < 0.05) of 16.65%, 11.37%, and 43.67% for temperatures of 50, 60, and 70 °C, respectively. There was a concentration of pectin with the increase in the dehydration temperature, with a statistical difference in both powder products as a function of the 20 °C increase (50–70 °C) with drying. Kpodo et al. [9], when evaluating the pectin contents of six genotypes of okra (*Abelmoschus esculentus* L.), isolated by aqueous extraction at a pH of 6.0, obtained values between 11% and 14% db.

As for the starch content, higher values were found for CM, with statistical differences (*p* < 0.05) at all temperatures under study (50, 60, and 70 °C), presenting a superiority of 372.05%, 290%, and 233.47%, respectively, in relation to OM. It was observed that, with the increase in drying temperature, there was an increase in starch, with a statistical difference between all temperatures in both powders. Starches are mimetic fat carbohydrates present in mucilages, cellulose, dextrins, and other fibers, and they can be used mainly as a thickener and stabilizer, acting on the smoothness and creaminess of emulsions [44]. They are of great importance for the application efficiency of the mucilages studied as drying aids.

The values of total soluble solids (TSS) of CM were statistically higher than those of OM by an average of 13.66%, with no statistical differences (*p* < 0.05) among drying temperatures. According to Capitani et al. [1], the considerable concentration of soluble solids in chia seeds is explained by the fact that the fraction of insoluble fiber present in the seeds is greater than that of soluble fiber, resulting in a high percentage of insoluble pectins, cellulose, hemicellulose, and lignin. A lower SST value was reported by Araújo et al. [17] with a content of 13.23 °Brix for the powdered mucilage of lyophilized seedless okra, obtained from extraction in a ratio of 1:2.5. It was observed that OM had a statically higher total sugar content than CM by 54.70% at a temperature of 50 °C, 43.83% at 60 °C, and 37.13% at 70 °C, verifying that, with the increase in drying temperature, there was a statistical reduction (*p* < 0.05) in the parameter in all studied powders. Faccio et al. [45], when analyzing the extract of lyophilized mucilage from jaracatiá (*Carica quercifolia* (A. St. Hil.) Hieron), found a total sugar content of 16.50% db corroborating with the OM values obtained. The reducing sugars showed behavior similar to the total sugars, with higher values for OM and a significant reduction with an increase in the drying temperature. This behavior is possibly due to the Maillard reaction, which culminates in the degradation of reducing sugars when complexed with free amino acids, synthesizing dark-colored products such as gums [46].

### 3.2. Powdered Mucilage Bioactive Compounds

Table 2 shows the bioactive compounds of powdered chia and okra mucilages dehydrated at different drying temperatures.

The powdered mucilage showed high levels of total phenolic compounds (TPC), with OM standing out with an average superiority of 48% in relation to CM (*p* < 0.05). Powdered mucilages showed a similar behavior with increasing drying temperature, with a significant concentration (*p* < 0.05) of phenolic compounds with increasing temperature. This behavior can be explained by the drying time, which is shorter at higher temperatures, in which the sample was exposed for less time to the drying air, thus reducing changes in the bioactive properties of the product [47]. Comparing the verified CFT contents for the extraction of raw materials, Jiang et al. [48], when dehydrating okra chips by different methods (oven at 70 °C, lyophilization, vacuum, and microwave), found average levels of phenolic compounds between 851.00 and 1159.00 mg EAG/100 g db. Alcântara et al. [49] obtained 609.60 mg EAG/100 g db for chia seed flour.

The average tannin contents were statistically higher (*p* < 0.05) in OM, showing high values in all drying temperatures, being almost twice the averages verified in CM. According to Costa et al. [50], the presence of hydrolyzable tannins is characteristic in vegetables with a high astringent power such as okra, being derived from gallic and ellagic acid, formed by sugar esters, which makes the material slightly palatable and reduces its nutritional power, while presenting considerable antioxidant capacity.

Anthocyanins were superior (*p* < 0.05) for OM at all temperatures. In both materials, an increase in average values was verified with the increase in drying temperature, with a 5.70% increase in CM and a 33.42% increase in OM between the lowest (50 °C) and the highest temperature (70 °C). According to Souza et al. [51], the higher concentration of this bioactive with the increase in drying temperature can be explained by the action of enzymatic browning, specifically caramelization, producing dark compounds that are detected in the total anthocyanin content.

In line with the other bioactives, the OM had higher average levels of flavonoids (*p* < 0.05); CM behaved similarly to OM, with an increase in average contents with drying temperature increasing. Nampuak and Tongkhao [52], studying the regeneration of powdered okra mucilage obtained by mixing whole okra and water followed by hydraulic pressing, addition of maltodextrin, and dehydration by spraying at 170 °C, verified a CFT content of 12.16 mg EAG/100 g db and flavonoid content of 5.43 mg catechin/100 g db when carrying out the regeneration in water in the ratio 120 mg/mL.

Beltrán-Orozco, Olguin, and Ramirez [53] confirmed the flavonoid content of 35.8 mg quercetin equivalent/100 g db for chia seeds. Gemede et al. [16] found a CFT content between 2466 and 4993 mg EAG/100 g db and total flavonoids ranging from 818 to 1872 mg EC/100 g db when observing okra powder mucilages extracted from pods of eight accessions obtained from the mixture of okra and water in the proportion 1:3, dehydrated in an oven at 45 °C.

**Figure 1:**
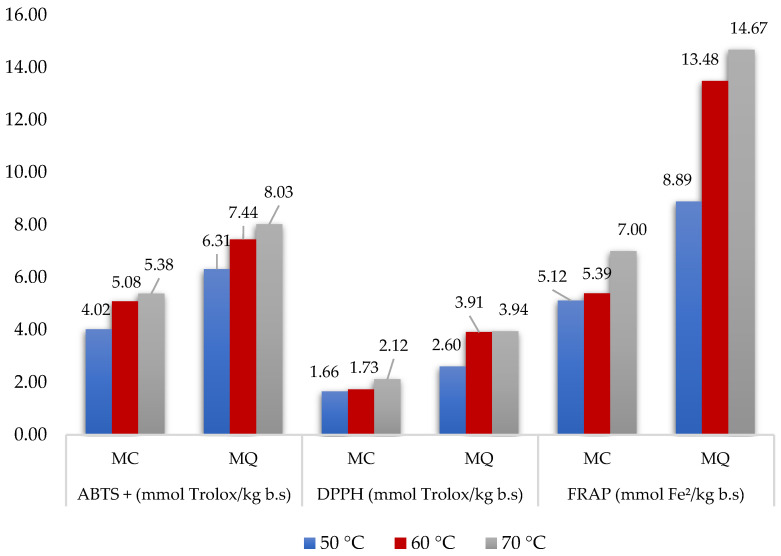
Antioxidant activity of chia (CM) and okra (OM) mucilage powder obtained at different drying temperatures determined by ABTS+, DPPH, and FRAP methods.

The antioxidant activity of powdered mucilages was determined by the ABTS+, DPPH, and FRAP radical assays; the results are shown in Figure 1.

It can be observed that OM presented the highest values in the three methodologies applied, indicating greater antioxidant activity. According to Martín-Gómez et al. [54], this relationship may be correlated with its higher content of total phenolic compounds, already shown in the previous table, since phenolic compounds are known to have excellent antioxidant properties, due to their ability to bind metal ions, reduce peroxides, and promote the potency of antioxidant enzymes [55]. Therefore, extracts with a higher content of total phenolic compounds have a greater antioxidant capacity due to the presence of hydroxyl and hydrogen atoms, showing greater effects of eliminating free radicals [56].

Regarding the influence of drying temperature on antioxidant activity, it was observed that, in all methods tested, the highest dehydration temperature (70 °C) influenced the conservation of antioxidants, showing the highest values. The increase in total phenolic compounds may result from the Maillard reaction (insertion of proteins reaction + sugars) that occurs in the process of dehydration by heating, with the production of new phenolic compounds and, consequently, increased antioxidant activity [57].

Evaluating the tests individually, it was verified that the FRAP method, which involves the direct measurement of antioxidants in a food product by the presence of reductions, presented the highest averages in both materials and in all studied temperatures, followed by the ABTS method; consequently, the analysis performed by DPPH inhibition presented the lowest values. Limmongkon et al. [58] suggested that there is no single assay to measure overall antioxidant capacity, as individual assays illustrate different aspects of antioxidant behavior. Therefore, the evaluation of generalized antioxidant capacity may need multiple assays to indicate the general antioxidant profile of plant extracts.

Gemede et al. [16], studying okra mucilage powder, extracted from the pods of eight accessions using a mixture of water in a 1:3 ratio before dehydrating in an oven at 45 °C, verified, using the DPPH methodology, an antioxidant activity ranging from 12.6 to 26.4 mmol Trolox/kg. Mujtaba et al. [59], producing chia mucilage nanocomposite films, found an antioxidant activity according to the FRAP assay of 484.94 mmol Fe^2+^/kg for the control composition.

### 3.3. Physical Characterization of Powdered Mucilages

Table 3 presents the average values of the physical parameters of the powdered chia and okra mucilages obtained at different drying temperatures.

The CM presented statistically higher solubility than OM, with an average superiority of 45.41%. Considering the solubility of commercial gums (xanthan, Arabic, guar, etc.), which are generally greater than 90% [60], powdered okra and chia mucilages showed low solubility. The low solubility of the mucilages studied compared to the commercial ones can probably be explained by the size of the powder particles; due to the rapid surface hydration capacity and the possibility of interaction between neighboring particles, it is possible that larger agglomerates form in which the smaller particles of polysaccharides offer less resistance to the hydration of their centers, forming a kind of gel that prevents the entry of water inside these agglomerates, such that the interior remains dry, making the dissolution process more difficult [61]. An indicated form of dissolution is through premixing the mucilage with other rapidly diluting material, such as salts and sugars, so that the mucilage particles are kept separated as much as possible, thus avoiding agglomeration. Ghori et al. [11] reported an average solubility of okra powder mucilages at different pH dried in an oven at 40 °C of 11 mg/g, concluding that the intrinsic viscosity of okra polysaccharides is largely dependent on the extraction protocol.

The mucilage showed low hygroscopicity, verifying higher averages in the OM (*p* < 0.05). Given these values, all powdered mucilages obtained at the studied drying temperatures were classified as having low hygroscopicity (<25.00%) [62]. It was proven that the increase in the drying temperature promoted a tendency to reduce the parameter, which may be related to the amorphous state of these powders; due to the increase in the drying temperature, there was probably a reduction in the amount of amorphous sugar and, consequently, a decrease in hygroscopicity [63]. Antigo et al. [15], observing powdered chia mucilage as an encapsulating agent for natural beet dye, in combination with maltodextrin and acacia gum (10 and 15%) in a spray dryer at 150 °C, found an average hygroscopicity of 10.01 to 12.22 g/100 g of dry powder.

The wettability rate of CM was lower than that of OM (*p* < 0.05), both presenting similar behaviors, with the parameter increasing with the increase in drying temperature. This behavior may be related to the influence of water content, with wettability being inversely proportional (i.e., the higher the water content, the lower the wettability of the powder, as well as the amount of pectin and starch present in the samples). Silva et al. [64], analyzing whole-meal flour and partially defatted bare flour (*Dipteryx alata* Vog.), found a higher wettability rate between 18.00 and 24.00 g/min.

Regarding the apparent density, OM with the highest average stood out, except for the mucilage obtained at a temperature of 50 °C, which did not statistically differ (*p* < 0.05). There was an inverse behavior between powdered mucilages; in CM, there was a tendency for density to decrease with increasing drying temperature, whereas, in OM, there was an increase in the parameter with increasing temperature. The apparent and compacted density is directly influenced by the material structure. In this case, the raw materials comprise a seed and a fruit; hence, the final result can be powders with different particles, porosity, and spaces between particles [65]. Gemede et al. [16] studying okra powdered mucilages extracted from the pods of eight accessions dried in an oven at 45 °C, found a variation in apparent density between 0.580 and 0.640 g/cm^3^. Timilsena et al. [36] found an apparent density of 0.189 g/cm^3^ for powdered chia mucilage, extracted in a 1:20 ratio and lyophilized, which is different from other commonly used food gums such as xanthan (0.650–0.850 g/cm^3^), guar gum (0.400 g/cm^3^), and arabic gum (0.400 g/cm^3^).

The compacted density of the mucilages comprises the characteristic of the powder when it reaches a state of greater equilibrium, i.e., an invariable packing arrangement directly linked to the structure of the particles and, consequently, to the capacity of flow and compression [66]. The compacted density of the powdered mucilages showed similar behavior to the apparent density. Similar values to the study were reported by Toit et al. [67] in lyophilized mucilage of three varieties of the cactus *Opuntia ficus-indica* (Algerian, Morado, and Gymno-Carpo) and the cultivar *Opuntia robusta* (Robusta), which verified an average apparent density between 0.560 and 0.590 g/cm^3^ and compacted density of 0.620 to 0.730 g/cm^3^.

The Carr indices (CI) or compressibility index of powdered mucilages showed increasing averages with increasing drying temperatures, but no statistical difference between mucilages at 50 and 70 °C (*p* < 0.05). The Carr index measures the flowability of powders. CI values of 15–20% indicate good fluidity, of 20–35% indicate poor fluidity, of 35–45% indicate poor fluidity, and >45% indicate very poor fluidity [33]. According to this criterion, it is verified that the mucilages in powder of both products can be considered as having good fluidity (CI < 20%). Okunlola, Odeniyi, and Arhewoh [68], studying okra powder mucilage dehydrated in an oven with air circulation at a temperature of 50 °C, as an encapsulant in ambroxol hydrochloride microspheres, also found good fluidity for IC powder mucilage of 14.65%.

The Hausner factor (FH) values, which correspond to the relation between the compacted and apparent densities and can be used to evaluate the cohesiveness of powdered products, presented an identical behavior to the Carr index, with a lower and similar value for the two types of dehydrated mucilages at a temperature of 50 °C (*p* < 0.05). According to Santhalakshmy et al. [34], powders that present Hausner factors lower than 1.2 are classified as having low cohesiveness, FH between 1.2 and 1.4 denotes intermediate cohesiveness, and FH >1.4 denotes high cohesiveness. Therefore, the powdered mucilages of both materials obtained at 50 °C can be classified as having low cohesiveness (FH = 1.14), while those obtained at temperatures of 60 and 70 °C (1.20 to 1.25) can be classified as having intermediate cohesiveness. Husain et al. [69], when carrying out a phytochemical study for the mucilage of *Althaea officinalis* L. root dehydrated at 50 °C in an oven, found a cohesiveness index of 1.17.

## 4. Conclusions

Powdered mucilage had low water content and water activity, as well as considerable pectin, starch, and protein contents. CM stood out in relation to water content, water activity, and hygroscopicity, showing lower averages and a reduction in contents with increasing drying temperature, as well as higher values of yield, ash, acidity, pectin, starch, total soluble solids, and solubility, with an increase in dehydration temperature.

OM, on the other hand, presented higher average contents of proteins and sugars, total phenolic compounds, tannins, anthocyanins, flavonoids, antioxidant activity, and wettability, showing an increase in their averages with the increase in drying air temperature.

As for the physical parameters, CM stood out in terms of higher solubility and lower hygroscopicity, while OM showed higher wettability rates. Both mucilages were classified as having good fluidity and low to intermediate cohesiveness.

The drying temperature’s influence on chemical, physical, and physicochemical properties of the mucilages was verified, obtaining better results for the temperature of 70 °C in both powdered products. Therefore, the study revealed that powdered chia and okra mucilages have good functional properties and can offer great potential as an industry raw material.

## 5. Patents

Patents resulting from the work reported in this manuscript are as follows:

− Chia mucilage (*Salvia hispanica*) powder–BR 10 2020 007595 0;
− Powdered mucilage of okra (*Abelmoschus esculentus* (L.) Moench)–BR 10 2020 008444 5.

## Figures and Tables

**Table 1:** Mean values and standard deviations of the chemical and physicochemical parameters of chia (CM) and okra (OM) mucilage powders obtained at different drying temperatures.

Parameters	Temperature (°C)	Powdered Mucilages	
		CM	OM
Yield (%)	50	21.04 ± 0.68 aA	13.40 ± 0.28 aB
	60	20.14 ± 0.39 aA	11.12 ± 0.99 bB
	70	17.06 ± 0.62 bA	9.73 ± 0.25 cB
Water content (% db)	50	6.05 ± 0.58 aB	8.90 ± 0.27 aA
	60	4.41 ± 0.09 bB	8.01 ± 0.41 bA
	70	2.55 ± 0.46 cB	6.68 ± 0.14 cA
Water activity (a_w_)	50	0.234 ± 0.002 aB	0.304 ± 0.005 aA
	60	0.179 ± 0.000 bB	0.259 ± 0.004 bA
	70	0.117 ± 0.003 cB	0.178 ± 0.001 cA
Ash (% db)	50	5.56 ± 0.06 aA	4.60 ± 0.07 aB
	60	5.47 ± 0.18 aA	4.83 ± 0.01 aB
	70	5.75 ± 0.34 aA	4.84 ± 0.23 aB
Total titratable acidity (% citric acid db)	50	1.37 ± 0.07 aA	0.89 ± 0.07 aB
	60	1.36 ± 0.08 aA	0.85 ± 0.08 bB
	70	1.31 ± 0.08 aA	0.84 ± 0.07 bB
pH	50	6.50 ± 0.02 cA	6.17 ± 0.01 bB
	60	6.94 ± 0.04 bA	6.19 ± 0.01 bB
	70	7.07 ± 0.02 aA	6.21 ± 0.01 aA
Proteins (% db)	50	18.83 ± 0.44 aB	24.27 ± 0.33 aA
	60	18.64 ± 0.13 aB	20.58 ± 0.47 bA
	70	18.83 ± 0.34 aA	17.99 ± 0.49 cB
Pectin (% db)	50	8.90 ± 0.19 bA	7.63 ± 0.70 bB
	60	9.01 ± 0.88 bA	8.09 ± 0.62 bB
	70	12.73 ± 0.74 aA	8.86 ± 0.43 aB
Starch (% db)	50	7.60 ± 0.05 cA	1.61 ± 0.02 cB
	60	8.19 ± 0.08 bA	2.10 ± 0.01 bB
	70	8.27 ± 0.06 aA	2.48 ± 0.02 aB
Total soluble solids (°Brix db)	50	18.56 ± 0.00 aA	16.33 ± 0.00 aB
	60	18.27 ± 0.00 aA	16.20 ± 0.00 aB
	70	18.46 ± 0.01 aA	16.02 ± 0.02 bB
Total sugars (g/100 g db)	50	10.56 ± 0.03 aB	23.31 ± 0.07 aA
	60	10.10 ± 0.08 bB	17.98 ± 0.02 bA
	70	9.45 ± 0.13 cB	15.03 ± 0.03 cA
Reducing sugars (g/100 g db)	50	4.33 ± 0.01 aB	6.29 ± 0.03 aA
	60	3.19 ± 0.01 bB	5.93 ± 0.02 bA
	70	2.77 ± 0.02 cB	4.34 ± 0.14 cA

Means followed by the same lowercase letters in columns and uppercase letters in rows do not differ statistically according to Tukey’s test at 5% probability.

**Table 2:** Mean values and standard deviations of bioactive compounds in powdered chia (CM) and okra (OM) mucilages obtained at different drying temperatures.

Parameters	Temperature (°C)	Powdered Mucilages	
		CM	OM
Total phenolic compounds (mg EAG*/100 g db)	50	804.31 ± 2.45 cB	1522.55 ± 5.21 cA
	60	821.86 ± 3.82 bB	1636.21 ± 6.11 bA
	70	845.91 ± 2.41 aB	1677.67 ± 3.27 aA
Tannins (mg EAT**/100 g db)	50	536.93 ± 3.37 bB	1019.10 ± 5.79 cA
	60	574.50 ± 13.04 aB	1088.91 ± 3.54 bA
	70	574.87 ± 2.19 aB	1165.98 ± 4.90 aA
Anthocyanins (mg/100 g db)	50	33.85 ± 0.09 bB	39.64 ± 0.14 cA
	60	35.13 ± 0.14 aB	44.06 ± 0.12 bA
	70	35.78 ± 0.97 aB	52.89 ± 0.32 aA
Flavonoids (mg/100 g db)	50	33.24 ± 0.73 bB	49.85 ± 0.21 bA
	60	35.94 ± 0.24 aB	48.42 ± 0.25 cA
	70	36.26 ± 0.98 aB	66.73 ± 0.06 aA

Means followed by the same lowercase letters in columns and uppercase letters in rows do not differ statistically according to Tukey’s test at 5% probability. *EAG, gallic acid equivalent; **EAT, tannic acid equivalent.

**Table 3:** Mean values and standard deviations of the physical parameters of mucilage powder from chia (CM) and okra (OM) obtained at different drying temperatures.

Parameters	Temperature (°C)	Powdered Mucilages	
		CM	OM
Solubility (% db)	50	50.43 ± 0.83 bA	26.32 ± 0.51 bB
	60	50.78 ± 1.41 bA	27.93 ± 2.30 abB
	70	54.69 ± 1.03 aA	30.86 ± 1.77 aB
Higroscopicity (% db)	50	15.11 ± 0.08 aB	19.92 ± 1.10 aA
	60	14.79 ± 0.71 aB	19.73 ± 0.45 aA
	70	13.85 ± 0.81 bB	17.79 ± 0.51 bA
Wettability rate (g/min)	50	1.03 ± 0.05 cB	1.42 ± 0.06 cA
	60	1.27 ± 0.01 bB	1.60 ± 0.11 bA
	70	1.90 ± 0.08 aA	1.95 ± 0.07 aA
Apparent density (g/cm^3^)	50	0.567 ± 0.052 aA	0.576 ± 0.008 bA
	60	0.492 ± 0.011 bB	0.623 ± 0.017 aA
	70	0.472 ± 0.003 bB	0.627 ± 0.011 aA
Compacted density (g/cm^3^)	50	0.656 ± 0.059 aA	0.643 ± 0.009 bA
	60	0.590 ± 0.003 bB	0.761 ± 0.019 aA
	70	0.588 ± 0.004 bB	0.779 ± 0.014 aA
Carr index (%)	50	12.00 ± 0.00 cA	12.00 ± 0.00 cA
	60	15.33 ± 2.31 bB	17.67 ± 0.56 bA
	70	19.67 ± 0.58 aA	20.00 ± 0.00 aA
Hausner factor	50	1.14 ± 0.00 cA	1.14 ± 0.00 cA
	60	1.20 ± 0.03 bA	1.21 ± 0.08 bA
	70	1.24 ± 0.01 aA	1.25 ± 0.00 aA

Means followed by the same lowercase letters in columns and uppercase letters in rows do not statistically differ according to Tukey’s test at 5% probability.

## Data Availability

Data can be digitized from the graphs or requested to the corresponding author.
